# Structure-Activity Relationships Based on 3D-QSAR CoMFA/CoMSIA and Design of Aryloxypropanol-Amine Agonists with Selectivity for the Human β3-Adrenergic Receptor and Anti-Obesity and Anti-Diabetic Profiles

**DOI:** 10.3390/molecules23051191

**Published:** 2018-05-16

**Authors:** Marcos Lorca, Cesar Morales-Verdejo, David Vásquez-Velásquez, Juan Andrades-Lagos, Javier Campanini-Salinas, Jorge Soto-Delgado, Gonzalo Recabarren-Gajardo, Jaime Mella

**Affiliations:** 1 Escuela de Quimica y Farmacia, Facultad de Medicina, Universidad Andres Bello, Quillota 980, Viña del Mar 2531015, Chile; m.lorcacarvajal@uandresbello.edu; 2 Centro de Nanotecnología Aplicada, Facultad de Ciencias, Universidad Mayor, Camino la Pirámide 5750, Huechuraba, Santiago 8580000, Chile; camoralv@uc.cl; 3 Facultad de Ciencias Químicas y Farmacéuticas, Universidad de Chile, Sergio Livingstone 1007, Independencia, Santiago 8380492, Chile; dvasquez@ciq.uchile.cl (D.V.-V.); jandrades@ug.uchile.cl (J.A.-L.); 4 Facultad de Ciencia, Universidad San Sebastián, Lago Panguipulli 1390, Puerto Montt 5501842, Chile; javier.campanini@uss.cl; 5 Departamento de Ciencias Quimicas, Facultad de Ciencias Exactas, Universidad Andres Bello, Quillota 980, Viña del Mar 2531015, Chile; jorge.soto@unab.cl; 6 Departamento de Farmacia, Facultad de Química, Pontificia Universidad Católica de Chile, Casilla 306, Avda. Vicuña Mackenna 4860, Macul, Santiago 7820436, Chile; grecabarren@uc.cl; 7 Centro de Investigación Farmacopea Chilena (CIFAR), Universidad de Valparaíso, Av. Gran Bretaña 1111, Valparaíso 2360102, Chile; 8 Instituto de Química y Bioquímica, Facultad de Ciencias, Universidad de Valparaíso, Av. Gran Bretaña 1111, Valparaíso 2360102, Chile

**Keywords:** β3-adrenergic receptor, obesity, diabetes, overactive bladder, aryloxypropanolamines, mirabegron, vibegron, 3D-QSAR, CoMFA, CoMSIA

## Abstract

The wide tissue distribution of the adrenergic β3 receptor makes it a potential target for the treatment of multiple pathologies such as diabetes, obesity, depression, overactive bladder (OAB), and cancer. Currently, there is only one drug on the market, mirabegron, approved for the treatment of OAB. In the present study, we have carried out an extensive structure-activity relationship analysis of a series of 41 aryloxypropanolamine compounds based on three-dimensional quantitative structure-activity relationship (3D-QSAR) techniques. This is the first combined comparative molecular field analysis (CoMFA) and comparative molecular similarity index analysis (CoMSIA) study in a series of selective aryloxypropanolamines displaying anti-diabetes and anti-obesity pharmacological profiles. The best CoMFA and CoMSIA models presented values of *r*^2^*_ncv_* = 0.993 and 0.984 and values of *r*^2^*_test_* = 0.865 and 0.918, respectively. The results obtained were subjected to extensive external validation (*q*^2^, *r*^2^, *r*^2^*_m_*, etc.) and a final series of compounds was designed and their biological activity was predicted (best pEC_50_ = 8.561).

## 1. Introduction

The β3 adrenergic receptor (β3-AR) is a transmembrane protein that belongs to the superfamily of G protein-coupled receptors [1,2]. There are three subtypes of β adrenergic receptors. The β1 adrenergic receptor is mainly located in the cardiovascular system, where it is the target of selective blockers such as atenolol or bisoprolol, which are used for the treatment of hypertension [3]. The β2 adrenergic receptor is mainly located in smooth muscles, where their activation by agonists such as salbutamol or salmeterol enables asthma treatment [4]. On the other hand, the β3-AR is widely distributed in the human body. It is present in the brain [5], the cardiovascular system [6], colon, bladder, and adipose tissue [7]. Therefore, it could be a therapeutic target for the treatment of diseases such as depression [8], hypertension, heart failure [9], overactive bladder (OAB) syndrome [10], colon cancer [11], metabolic syndrome, and obesity [12].

**Figure 1:**
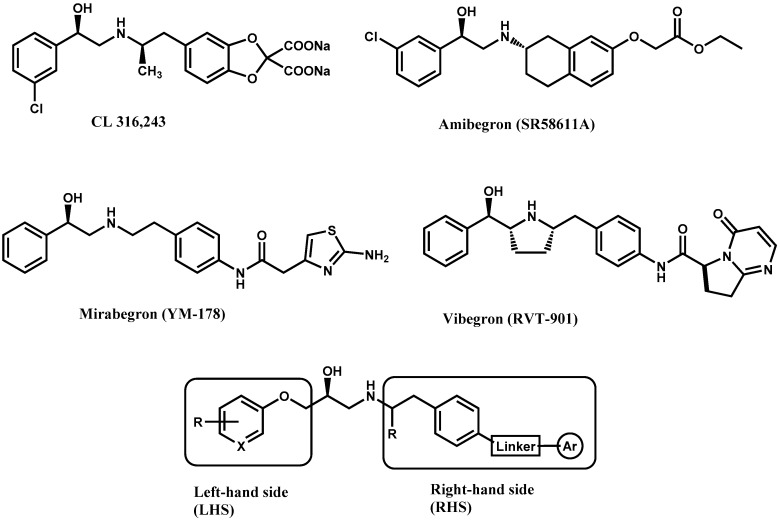
Structures of CL 316,243, amibegron, mirabegron, vibegron and the general structure of the aryloxypropanolamines studied here.

Until now, the pharmacophore for the design and synthesis of new β3-AR ligands has been the ethanolamine chain. Most of the compounds are of the phenylethanolamine or aryloxypropanol-amine type. To achieve β3 adrenergic selectivity, the insertion of bulky groups on the right-hand side (RHS) of the molecule is favorable (Figure 1). However, since the approval of mirabegron in 2012, few selective agonist compounds for the β3-AR receptor have been reported [13,14,15]. Selective β3 adrenergic agonists include CL 316,243 [16], amibegron (SR58611A) [17,18], mirabegron (YM-178) [10], and vibegron (RVT-901) [19] (Figure 1). CL 316,243 has anti-obesity and anti-diabetic profiles [20]. Amibegron presents antidepressant effects in animal models [21]. Mirabegron is the only selective β3 drug currently approved by the U.S. Food and Drug Administration for the treatment of OAB syndrome [22], however, there have been reports of upper airway angioedema following the administration of mirabegron [23]. On the other hand, recent studies have shown that mirabegron raises blood pressure and prolongs the QTc interval in electrocardiograms [24]. This information calls into question the continuity of mirabegron in the market. In this scenario, Merck Laboratories reported in 2016 the discovery of vibegron (Figure 1), a new potent and selective β3-AR agonist, which is currently under development in human clinical trials for the treatment of OAB [19].

From the works of Cramer and Klebe [25,26], comparative molecular field analysis (CoMFA) and comparative molecular similarity index analysis (CoMSIA) are considered useful methodologies to understand the pharmacological properties of a series of compounds. Contour maps generated from CoMFA and CoMSIA show regions of the molecular structure where modifications in the steric, electrostatic, hydrophobic, and H-bond properties generate a favorable or unfavorable change in biological activity. Therefore, the contour maps obtained help to: (a) understand the nature of ligand-receptor interactions; (b) predict biological activity; and (c) aid in the rational design of new compounds.

In the last 10 years, there have been only two reports of quantitative structure-activity relationship (QSAR) studies on selective compounds for the β3-AR [27,28], one of which was conducted by our research group [28]. In both cases, the studies were carried out on phenylethanolamine-type compounds. Since then, there have been no reports of QSAR studies on aryloxypropanolamines. In the present work, we present a three-dimensional (3D)-QSAR study of a series of potent and selective human β3-AR agonists [29,30]. The reported compounds showed an interesting profile as potential drugs for the treatment of obesity and noninsulin-dependent (type II) diabetes. The compounds have a wide structural variability on both the RHS and left-hand side (LHS) of the general aryloxypropanolamine structure (Figure 1). The information obtained from the CoMFA and CoMSIA contour maps was systematized in a useful structural-activity relationship diagram. With this information, we finally reported the design of new compounds with promising β3 adrenergic activity.

## 2. Results and Discussion

### 2.1. Statistical Results

A summary of the statistical results for CoMFA and CoMSIA are presented in Table 1. Details of all possible combinations are given in Table S1 (Supplementary Material). 

The best models were searched through successive field combinations. The first parameter to evaluate the statistical robustness of a QSAR model is the value of *q*^2^, which must be greater than 0.5. *q*^2^ is an indicator of the internal predictive capacity of a QSAR model. For CoMFA, the model that considered both field contributions (CoMFA-SE) presented the highest value (0.537), while with CoMSIA, several models presented a significant *q*^2^ value. The CoMSIA steric + electrostatic + hydrophobic + acceptor (CoMSIA-SEHA) and CoMSIA steric + electrostatic + acceptor (CoMSIA-SEA) models generated similar values for this coefficient (0.674 and 0.651, respectively). However, the value of *r*^2^, which evaluates the external predictive capacity of the model, allowed for discrimination between the models. In this case, the CoMSIA model that considered all the field contributions (CoMSIA-All) presented the highest value of *r*^2^ (0.918). The best models also had a low SEE and a high *r*^2^. The optimal number of components (*N*) is also low in all models presented (*N* = 6 for the best CoMFA and CoMSIA model). Ideally, a good model should have as few components as possible (*N* should be less than one-third of the total number of compounds studied), which ensures that the predictions will be based on meaningful information from field contributions, rather than on overtraining of the model. There is also a balance in the percentages of field contribution (approximately 50% for each field in CoMFA-SE and approximately 20% for each field in CoMSIA-All), which supports the reliability of the conclusions obtained from each contour map.

Table 2 presents a summary of the external validation of the CoMFA-SE and CoMSIA-All models (hereafter they are referred to as “CoMFA” and “CoMSIA” models). Both models have a high value for *r*^2^ (0.865 and 0.918, respectively), which is an indication of an adequate external predictive capacity. However, according to Golbraikh and Tropsha [31,32], high values of *q*^2^ and *r*^2^ (conditions 1 and 2) are necessary but not sufficient conditions for the validation of a model. For a QSAR model to have a reliable predictive capability, the line for experimental versus predicted activity should be as close as possible to the line *y* = *x*. This is observed in the fulfillment of conditions 3a or 3b, 4a or 4b, 5a or 5b, and 6 listed in Table 2. Finally, condition 7, known as *r*^2^*_m_* metrics, is a quantitative measure to determine the proximity between the observed and the predicted activity for the test set. The CoMFA and CoMSIA models reported here fulfilled all the conditions for internal and external validation and, in general, the CoMSIA model displays better statistical parameters than CoMFA.

**Figure 2:**
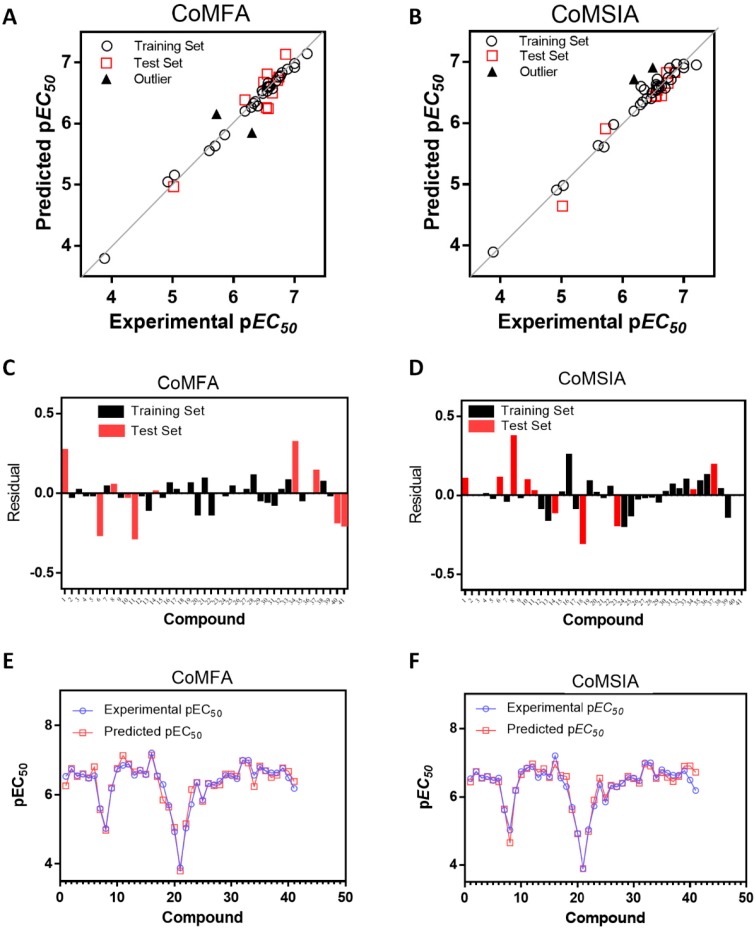
Plots of experimental versus predicted pEC_50_ values for the training and test set molecules for CoMFA (**A**) and CoMSIA (**B**) models. Residual plots between predicted and experimental values for CoMFA (**C**) and CoMSIA (**D**); CoMFA (**E**) and CoMSIA (**F**) predictions for every molecule in the complete set.

The values of experimental activity, predicted activity, and residual values for the best CoMFA and CoMSIA models are shown in Table 3. All the compounds showed low residual values and deviations in the predicted activity over a logarithmic unit were not observed. Figure 2A,B show the graphs of experimental versus predicted activity, from which it can be seen that the data distribution is close to the *y* = *x* line. Both models show a good balance in terms of predictive capacity. The CoMFA model presents 21 compounds with negative residual values and 20 with positive deviations (Figure 2C), while the CoMSIA model presents 19 compounds with negative residual values and 22 with positive deviations (Figure 2D). The residual ranges were −0.44 to 0.45 for CoMFA and −0.53 to 0.37 for CoMSIA. As shown in Figure 2E,F, the CoMFA and CoMSIA models show a satisfactory predictive capability throughout the whole set of data (training and test set) as well as a good predictive power for both, less active (**8**, **20**, and **21**) and most active compounds (**16**, **32**, and **33**).

Furthermore, to assess the robustness of the model, the Y-randomization test [33] was applied (see Table S2 of the Supplementary Material for randomizations). The dependent variable (biological activity) was randomly shuffled and a new QSAR model was developed using the original independent variable matrix. If after multiple randomizations the new values of *q*^2^ and *r*^2^*_ncv_* are negative or below the limit of acceptability (*q*^2^ < 0.5, *r*^2^*_ncv_* < 0.6), then it is corroborated that the results obtained in the formulation of the final models are not by chance. In our case, the new QSAR models (after several repetitions) have low *q*^2^ and *r*^2^*_ncv_* values (Table 4).

In summary, the best CoMFA and CoMSIA models were selected based on their statistical robustness and good validated external predictability. In the case of CoMFA, both potentials contribute equally to biological activity (41.2% for the steric field and 58.8% for the electrostatic field). In the case of CoMSIA there is a homogeneous contribution of each field to the activity, however, the electrostatic field presents a slightly higher contribution (27.9%), so its contribution to biological activity is more important.

### 2.2. Contour Maps Analysis

#### 2.2.1. CoMFA

**Figure 3:**
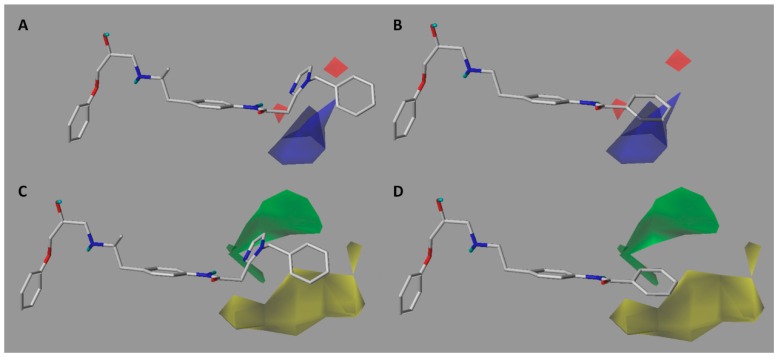
CoMFA electrostatic (**A**,**B**) and steric (**C**,**D**) contour maps around compounds **16** (**left**) and **21** (**right**), the most active and less active of the series respectively. Electropositive favored (blue) and electronegative favored (red). Sterically favored (green) and disfavored (yellow).

In the electrostatic contour map (Figure 3A), we can see a blue polyhedron around the methylene connector that connects the amide with imidazole ring. This suggests that the replacement of this connector by electropositive groups would be favorable for activity. For example, groups such as CONH, CO(NH)_2_, or a protonated amino group (projecting the proton towards the blue polyhedron) could be evaluated. This could explain why compounds **32** and **33** are among the most active since they project the polar proton of the urea function towards the blue zone, while compounds **8** and **20** present low activities due to the absence of said function. On the other hand, as seen in Figure 3B, the most inactive compound of the series (compound **21**) intersects the blue polyhedron. Therefore, one way to improve the activity of this type of derivative would be to increase the electronic deficiency of the benzene ring. This could be achieved by inserting electronic attractor groups into the ring or by inserting groups such as OH or NH that project the hydrogen atom to the blue region.

In the steric contour map around the most active compound **16** (Figure 3C), a green polyhedron is seen around position 5 of the imidazole ring. Therefore, the insertion of bulky substituents in that position would be favorable. This could explain the high activities reported for compounds **12** and **32**, which project a benzyl and nitro group, respectively, to the green region. In Table 5, the proposals **1x** to **5x** were based on this observation. These compounds contain OH, NH, F, and acetyl groups in that position. The use of more voluminous halogens did not generate better predictions (e.g., Cl, Br, or I). On the other hand, in the less active compounds **7**, **20**, and **21** (Figure 3D), the benzene ring intersects the yellow region, which could explain the low activity of these compounds.

#### 2.2.2. CoMSIA

**Figure 4:**
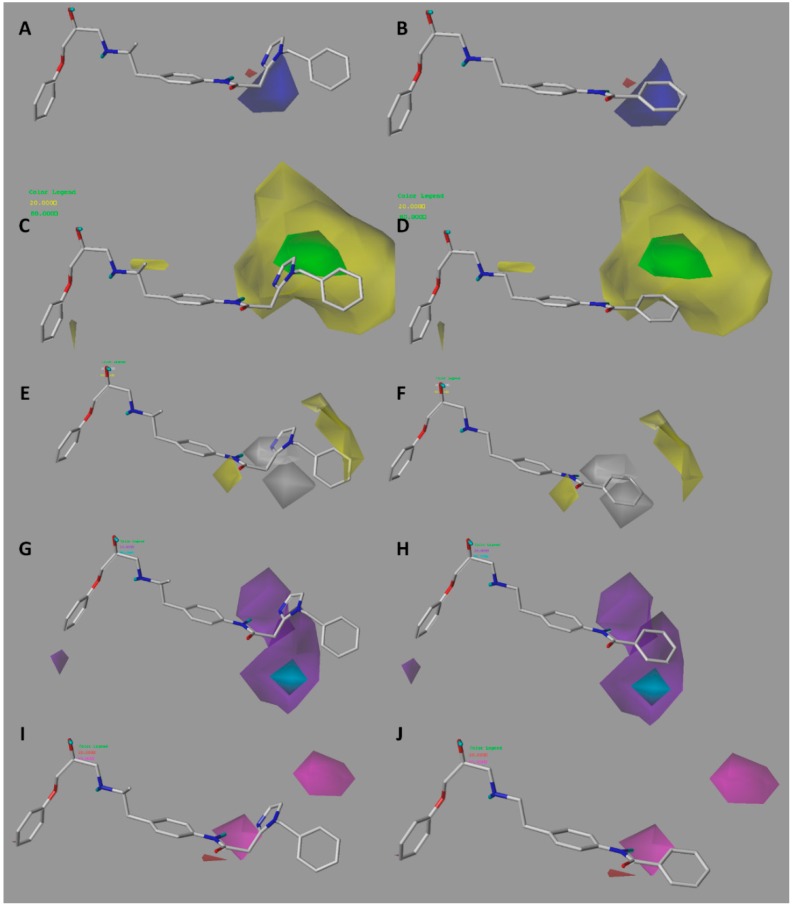
CoMSIA electrostatic (**A**,**B**); steric (**C**,**D**); hydrophobic (**E**,**F**); donor (**G**,**H**) and acceptor (**I**,**J**) contour maps around compounds **16** (**left**) and **21** (**right**), the most active and less active of the series respectively. The colors in **A**–**D** have the same meaning as in the CoMFA contour maps. Hydrophobic favored areas are in yellow and unfavorable areas in grey (**E**,**F**). Donor and acceptor favored areas are in cyan and magenta respectively, and donor and acceptor unfavorable areas are in purple and red, respectively (**G**–**J**).

The CoMSIA electrostatic contour map (Figure 4A,B) is similar to that obtained for CoMFA (Figure 3A,B). A blue polyhedron is visible near the methylene linker (Figure 4A). The concordance of this information in both models led us to propose the insertion of a urea group, after which we obtained derivatives with high predicted activity (Table 5). As in CoMFA, the less active compound **21** intersects the blue polyhedron in the benzene region, therefore the insertion of electropositive functions before the ring would be most favorable for activity.

Like the CoMFA map (Figure 3C), the steric contour map shows a green region intersecting position 5 of the imidazole ring of compound **16** (Figure 4C). However, on the CoMSIA map, a yellow region surrounds the green region, therefore the increase in volume should be explored with caution. In fact, in the proposal for new structures, it was found that the insertion of large groups in position 5, such as Br and Cl, generated a reduction in biological activity, however, the insertion of medium-volume groups such as OH, NH_2_, and F improved activity considerably. This also suggests that the increase in molar refractivity is not favorable for activity.

The hydrophobic contour map (Figure 4E,F) shows two yellow polyhedrons, one near the carbonyl oxygen and the other near the benzene ring. This indicates that the presence of lipophilic groups in these regions would be favorable for activity. The high activity reported for compounds **32**, **33**, **35**, and **39** could be explained by this fact since they position the sulfur atom of the sulfonylurea linker towards the smaller yellow polyhedron. In addition, in those same derivatives, the proton of the NH group at the right of the connector is directly positioned towards a grey polyhedron, which suggests that the presence of hydrophilic groups in that area is favorable. On the other hand, around the most active compound **16**, there is a second grey polyhedron intersecting the imidazole ring (Figure 4E), which implies that this ring could be replaced by other hydrophilic isostere rings, but not by systems such as benzene or thiophene. Finally, the second yellow polyhedron intersects the benzene ring of the most active compound **16**, but not the benzene ring of the least active compounds **19**–**23** (Figure 4F), which may in part explain the lower activity observed for these derivatives.

A large purple polyhedron surrounding the imidazole ring and the ortho position of benzene around the most active compound **16** (Figure 4G) is shown on the H-bond donor map (Figure 4G,H), suggesting that the presence of H-bond donor groups in these positions is not favorable for activity. This may explain the low activity of compound **8** (which is among the series of compounds **5**–**18**) because it directly positions the NH group of the imidazole ring to the upper purple region. On the other hand, a smaller cyan polyhedron in the lower area suggests that the selective insertion of H-bond donor groups in the methylene connector area of imidazole would be beneficial. This is corroborated by the fact that the most active compounds **32**, **33**, and **35** position the NH group of the sulfonylurea towards this polyhedron. Finally, a small purple polyhedron on the LHS of the molecule suggests that the presence of NH groups of the dihydrobenzimidazolone and indole ring systems would not be beneficial for activity.

The H-bond acceptor contour map (Figure 4I,J) shows two magenta polyhedra close to position 3 and 5 of the imidazole ring, which means that incorporation of H-bond acceptor atoms in these positions is favorable. In fact, compounds **32** and **33** position the oxygen atoms from nitro groups to the magenta region. Other groups that could be inserted are F, OH, and pyridine rings.

### 2.3. Outliers

In the CoMFA model, compounds **18** and **23** were outliers. Compound **18** has a pEC_50_ = 6.3010 and unlike its analog **16** (the most active compound in the series), it has an alkylation in the *N* of the amide. Therefore, the spatial conformation of the imidazole ring may be altered. On the other hand, alkylation of the ethyl chain in compound **18** restricts rotation and could fix a different conformation within the target. With respect to the underestimation of activity for compound **23**, this may be because CoMFA does not consider the favorable effects of the presence of the urea group. Effects that are considered by the hydrophobic and H-bond acceptor maps of CoMSIA, where compounds **18** and **23** were not outliers.

The outlier compounds in CoMSIA were **40** and **41**, for which the model predicts greater activity than the real one. This imprecision may be because, in the case of compound **40**, it positions a sulfonamide group towards the magenta polyhedral of the H-bond acceptor map, which is favorable for activity. However, this group falls into the yellow region of the steric map, but the greater contribution of the H-bond acceptor potential to the activity overestimates the predicted activity. In the case of compound **41**, the overestimation of the biological activity value may be due to the reduction in the electronic density of the benzene ring, given by the thiourea group, which has the highest percentage of contribution to biological activity.

### 2.4. Applicability Domain

The applicability domain (AD) is a theoretical region in chemical space encompassing both the model descriptors and modeled response, which allows one to estimate the uncertainty in the prediction of a compound based on how similar it is to the training compounds employed in the model development. In this work, we used the method developed by Roy et al. [34] for the determination of AD. This method is based on the basic theory of the standardization approach.

The calculation was carried out using the free application available on the author’s page, after which it was obtained that all compounds were within the domain of applicability, except compound **18**. This reinforces what was described in the previous section, that alkylation of the urea connector could result in significant changes in receptor binding. For this reason, none of the designed compounds (see next section) included alkyl groups in the urea connector.

### 2.5. Design of Novel Derivatives

Based on the information provided by CoMFA and CoMSIA, we have designed a series of structures of the aryloxypropanolamine type. In Table 5, we present the best derivatives with their predicted pEC_50_ value by the best model (CoMSIA, *r*^2^ = 0.918). The first proposed molecule **1x** was as active as compound **16** (pEC_50_ = 7.208). The other structures (**2x**–**12x**) are more active than compound **16**. The best candidates are compounds **3x** (pEC_50_ = 8.561) and **7x** (pEC_50_ = 8.520). The presence of polar functions like OH, NH_2,_ and F at position 5 of the imidazole ring yielded good candidates. Other interesting proposals are the replacement of imidazole with a benzimidazole ring (comp. **9x**–**12x**), in which the presence of polar functions improves the activity.

## 3. Materials and Methods

### 3.1. Selection of Conformers and Molecular Alignment

**Figure 5:**
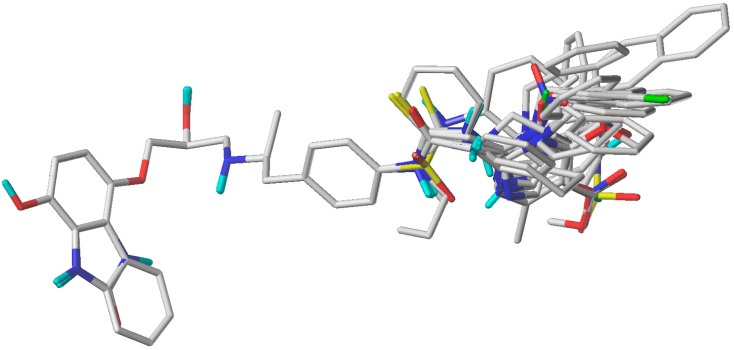
The superimposed structures of all compounds used in the CoMFA/CoMSIA models.

CoMFA and CoMSIA studies were performed with Sybyl X-1.2 software (1.2, Tripos International, St. Louis, MS, USA) installed in a Windows 10 environment on a PC with an Intel Core i7 CPU. To acquire the best conformers for each molecule, every compound was drawn in ChemDraw and subjected to a preliminary geometry optimization using MM2 molecular mechanics as is implemented in ChemBio3D software (15.1.0, PerkinElmer, Waltham, MA, USA). Following this, the structure of compound **16** (the most active of the series) was further minimized by quantum mechanics using the DFT B3LYP/6.311+g** method in Gaussian software (09, Gaussian Inc., Wallingford, CT, USA). This structure was used as a template for the alignment. The mol2 structures were imported to Sybyl and MMFF94 charges were assigned to each atom. The minimized structures were superimposed by the atom-by-atom fit method choosing the aryloxypropanolamine nucleus as the common scaffold for alignment (Figure 5). In addition, a minimization was carried out based on the Powell method [35] (as implemented in Sybyl, Figure S1 in the Supplementary Material). However, the statistical results were much lower than those reported by the method used in this manuscript (Table S3 in the Supplementary Material).

### 3.2. CoMFA and CoMSIA Field Calculation

To derive the CoMFA and CoMSIA descriptor fields, the aligned training set molecules were placed in a three-dimensional cubic lattice with a grid spacing of 2 Å in the *x*, *y*, and *z* directions such that the entire set was included in it. The CoMFA steric and electrostatic field energies were calculated using an sp^3^ carbon probe atom with a van der Waals radius of 1.52 Å and a charge of +1.0. Cut-off values for both steric and electrostatic fields were set to 30.0 kcal/mol. For CoMSIA analysis, the standard settings (probe with charge +1.0, radius 1 Å, hydrophobicity +1.0, H-bond donating +1.0, and H-bond accepting +1.0 [26]) were used to calculate five different fields: steric, electrostatic, hydrophobic, donor, and acceptor. Gaussian-type distance dependence was used to measure the relative attenuation of the field position of each atom in the lattice and led to a much smoother sampling of the fields around the molecules when compared to CoMFA. The default value of 0.3 was set for attenuation factor α.

### 3.3. Data Set Selection and β3-Adrenergic Activity

**Figure 6:**
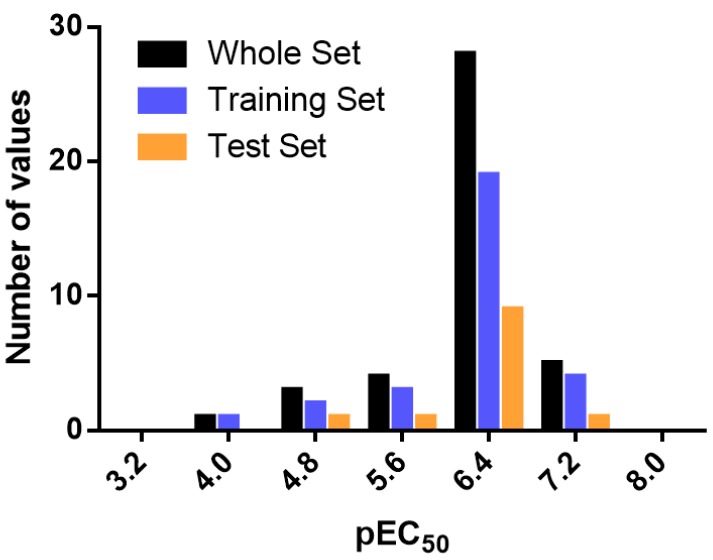
Histogram of frequency distribution data.

CoMFA and CoMSIA studies were performed on a set of 41 phenoxypropanolamine derivatives reported by Astellas Pharma [29,30] (Table 6). The derivatives displayed potent agonistic activity at the β3-AR. Agonistic activity (EC_50_) was assessed by measuring cAMP accumulation in CHO cells expressing β3-ARs. The EC_50_ values were converted to pEC_50_ (−logEC_50_). Several combinations of training and test sets were evaluated. The compounds were manually and randomly divided into training (29 compounds, 70%) and test sets (12 compounds, 30%), ensuring that both sets contained structurally diverse compounds with high, medium, and low activity, and a uniform distribution to avoid possible problems during external validation. For this purpose, most of the test set compounds were randomly extracted from the range of 6–8 logarithmic units of pEC_50_, while a smaller number were randomly selected from the range of 4–6 logarithmic units. Several test set groups were evaluated. For the construction of the final models, the test set that generated the highest *r*^2^ value in each case (CoMFA and CoMSIA) was finally selected. The distribution of pEC_50_ values for the whole set, the training set, and the test set is shown in Figure 6. In all three cases, the biological activity followed a Gaussian distribution. The range of the biological activities spans 3.5 log units, from 3.89 to 7.37.

### 3.4. Internal Validation and Partial Least Squares (PLS) Analysis

PLS analysis was used to construct a linear correlation between the CoMFA and CoMSIA descriptors (independent variables) and the activity values (dependent variables) [36]. To select the best model, the cross-validation analysis was performed using the leave-one-out (LOO) method (and sample distance PLS [SAMPLS]), which generates the square of the cross-validation coefficient (*q*^2^) and the optimum number of components (*N*). The non-cross validation was performed with a column filter value of 2.0 to speed up the analysis and reduce the noise. The *q*^2^, which is a measure of the internal quality of the models, was obtained according to the following Equation (1):

(1) $$q^{2}=1-\frac{\sum {\left(y_{i}-y_{pred}\right)}^{2}}{\sum {\left(y_{i}-\bar{y}\right)}^{2}}$$

where $y_{i}$, $\bar{y}$, and $y_{pred}$ are observed, mean, and predicted activity in the training set, respectively.

### 3.5. External Validation

The models were subjected to external validation criteria according to the proposed test by Golbraikh and Tropsha [31,32], which considers a QSAR model predictive, if the following conditions are satisfied:

(2) $$q^{2}>0.5$$

(3) $$r^{2}>0.6$$

(4) $$\frac{\left(r^{2}-r_{0}^{2}\right)}{r^{2}}<0.1\;\mathrm{or}\;\frac{\left(r^{2}-r_{0}^{\prime 2}\right)}{r^{2}}<0.1$$

(5) 0.85≤k≤1.15 or 0.85≤ k’≤1.15

(6) $$\left|r_{0}^{2}-{r^{\prime }}_{0}^{2}\right|<0.3$$

It has been demonstrated [31] that all of the above criteria are indeed necessary to adequately assess the predictive ability of a QSAR model. 

Furthermore, the external predictive power of the developed 3D-QSAR models using the test set was examined by considering $r_{m}^{2}$ metrics as shown below [37]:

(7) $$r_{m}^{2}=r^{2}\left(1-\left|\sqrt{r^{2}-r_{0}^{2}}\right|\right)$$

where $r^{2}$ and $r_{0}^{2}$ are the squared correlation coefficients between the observed and predicted activities of the test set with and without the (0,0) intercept, respectively. For a significant external model validation, the value of $r_{m}^{2}$ should be greater than 0.5.

### 3.6. Applicability Domain Calculation

The AD was evaluated based on the simple standardization method reported by Roy et al. [34]. First, each descriptor “*i*” for each compound “*k*” is standardized (*S_ik_*). Every compound must have a maximum value [*S_i_*]*_max_*_(*k*)_ ≤ 3. In the case that [*S_i_*]*_max_*_(*k*)_ > 3 and its minimum value [*s_i_*]*_min_*_(*k*)_ < 3, then the *S_new_*_(*k*)_ parameter must be calculated and has to fulfill the condition: *S_new_*_(*k*)_ = ${\bar{S}}_{k}+1.28\times \sigma_{S_{k}}$, where ${\bar{S}}_{k}$ is the mean of *S_ik_* values for compound *k* and $\sigma_{S_{k}}$ is the standard deviation for such values. The software is available free of charge on the authors’ website: http://dtclab.webs.com/software-tools and http://teqip.jdvu.ac.in/QSAR_Tools/.

## 4. Conclusions

In the present article, we presented the construction of two QSAR models in aryloxypropanolamines with selective potency for the β3-AR. The CoMFA and CoMSIA models presented good internal (*q*^2^ = 0.537 and 0.669 for CoMFA and CoMSIA, respectively) and external (*r*^2^ = 0.865 and 0.918 for CoMFA and CoMSIA, respectively) validation. The models were further validated following the criteria given by Tropsha and Roy [31,32,37], and were determined to be statistically reliable and robust. In both models, there was an equilibrium among the steric, electrostatic, hydrophobic, H-bond acceptor, and H-bond donor contribution to the activity. With this information, a new series of compounds was designed. The predicted biological activity for the new derivatives is high and, in general, the presence of polar groups and cycles like the benzimidazole ring on the RHS are predicted to improve activity. This could be due the presence of polar functions may be important for interaction with the Arg315 residue, and aromatic rings may establish pi-stacking interactions with a Phe198 residue as it is reported in literature [38].

**Figure 7:**
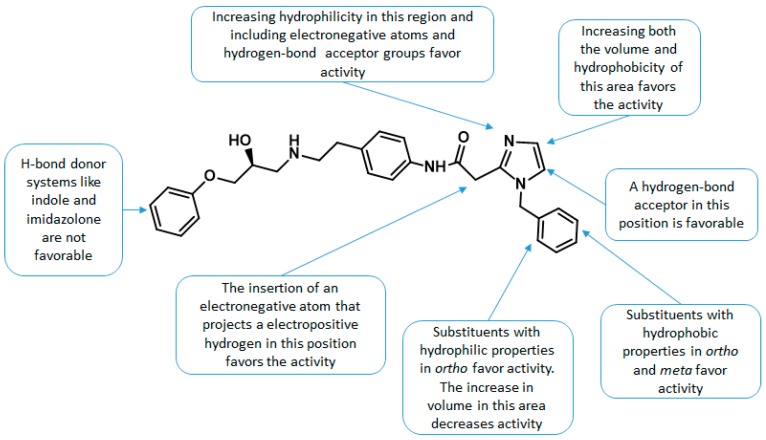
Main structure-activity relationships found in this study.

Taking into account the information derived from the CoMFA and CoMSIA studies, we have summarized the principal structure-activity relationships for the studies series of compounds in Figure 7. This information will be useful for the design of new compounds with promising therapeutic applications in several pathological disorders such as obesity, diabetes, OAB, depression, and cancer.

## Figures and Tables

**Table 1:** Statistical parameters and Field combinations for CoMFA and CoMSIA ^a^.

Model	*q* ^2^	*N*	*SEP*	*SEE*	*r* ^2^ *_ncv_*	*F*	*r* ^2^	Field Contributions				
								S	E	H	D	A
CoMFA-SE	0.537	6	0.544	0.067	0.993	525.4	0.865	0.412	0.588			
CoMSIA-SE	0.566	7	0.539	0.101	0.985	193.0	0.002	0.299	0.701			
CoMSIA-SEHA	0.674	6	0.456	0.119	0.978	161.3	0.790	0.174	0.335	0.215		0.276
CoMSIA-SEA	0.651	5	0.462	0.151	0.963	118.7	0.760	0.245	0.395			0.360
CoMSIA-SEDA	0.601	7	0.517	0.103	0.984	185.5	0.816	0.229	0.324		0.190	0.257
CoMSIA-SD	0.551	6	0.536	0.217	0.926	46.2	0.347	0.470			0.530	
CoMSIA-SHD	0.561	9	0.570	0.095	0.988	172.1	0.237	0.312		0.398	0.289	
CoMSIA-EHA	0.598	6	0.507	0.136	0.971	123.0	0.765		0.427	0.299		0.274
CoMSIA-EHDA	0.508	7	0.574	0.111	0.982	160.3	0.716		0.375	0.261	0.156	0.208
CoMSIA-ALL	0.669	6	0.460	0.101	0.984	225.9	0.918	0.165	0.279	0.181	0.159	0.215

^a^*q*^2^ = the square of the leave-one-out (LOO) cross-validation (CV) coefficient; *N* = the optimum number of components; *SEP* = standard error of prediction; *SEE* is the standard error of estimation of non-CV analysis; *r*^2^*_ncv_* is the square of the non CV coefficient; *F* is the *F*-test value; *r*^2^ is the predictive *r*^2^ for test set compounds; S, E, H, D and A are the steric, electrostatic, hydrophobic, hydrogen-bond donor, and hydrogen-bond acceptor contributions respectively.

**Table 2:** Summary of external validation parameters for CoMFA and CoMSIA.

Condition	Parameters	Threshold Value	CoMFA	CoMSIA
1	$q^{2}$	>0.5	0.537	0.669
2	$r^{2}$	>0.6	0.865	0.918
3a	$r_{0}^{2}$	Close to value of $r^{2}$	0.864	0.911
3b	${r^{\prime }}_{0}^{2}$	Close to value of $r^{2}$	0.834	0.885
4a	k	0.85 < k < 1.15	1.002	0.996
4b	k′	0.85 < *k*′ < 1.15	0.937	1.004
5a	$\left(r^{2}-r_{0}^{2}\right)/r^{2}$	<0.1	0.001	0.007
5b	$\left(r^{2}-{r^{\prime }}_{0}^{2}\right)/r^{2}$	<0.1	0.036	0.034
6	$\left|r_{0}^{2}-{r^{\prime }}_{0}^{2}\right|$	<0.3	0.031	0.027
7	$r_{m}^{2}$	>0.5	0.793	0.843

*q*^2^ and *r*^2^ are the same parameters as listed in Table 1; $r_{0}^{2}$ and *k* are the correlation coefficient between the experimental (*x*) versus predicted activities (*y*) for test set through the origin and the respective slope of regression; and $r_{0}^{\prime 2}$ and *k*′ are the correlation coefficient between the predicted (*y*) versus experimental activities (*x*) for test set through the origin and the respective slope of regression. $r_{m}^{2}$ was defined in Equation (6).

**Table 3:** Experimental and predicted pEC_50_ and residual values for analyzed compounds according to CoMFA and CoMSIA.

		CoMFA		CoMSIA	
Molecule	Experimental pEC_50_	Predicted pEC_50_	Residual	Predicted pEC_50_	Residual
**1** ^t^	6.538	6.263	0.27	6.435	0.10
**2**	6.745	6.760	−0.02	6.743	0.00
**3**	6.553	6.532	0.02	6.552	0.00
**4**	6.602	6.610	−0.01	6.596	0.01
**5**	6.482	6.494	−0.01	6.496	−0.01
**6** ^t^	6.553	6.811	−0.26	6.444	0.11
**7**	5.602	5.559	0.04	5.635	−0.03
**8** ^t^	5.018	4.969	0.05	4.646	0.37
**9**	6.187	6.205	−0.02	6.197	−0.01
**10** ^t^	6.745	6.761	−0.02	6.651	0.09
**11** ^t^	6.854	7.136	−0.28	6.830	0.02
**12**	6.886	6.893	−0.01	6.965	−0.08
**13**	6.569	6.667	−0.10	6.721	−0.15
**14** ^t^	6.721	6.707	0.01	6.826	−0.10
**15**	6.585	6.601	−0.02	6.569	0.02
**16**	7.208	7.144	0.06	6.953	0.26
**17**	6.553	6.530	0.02	6.632	−0.08
**18** ^t,a^	6.301	5.853	0.45	6.601	−0.30
**19**	5.699	5.636	0.06	5.613	0.09
**20**	4.921	5.049	−0.13	4.908	0.01
**21**	3.886	3.794	0.09	3.894	−0.01
**22**	5.032	5.157	−0.13	4.980	0.05
**23** ^t,a^	5.721	6.157	−0.44	5.908	−0.19
**24**	6.357	6.362	−0.01	6.549	−0.19
**25**	5.854	5.818	0.04	5.979	−0.13
**26**	6.328	6.331	0.00	6.345	−0.02
**27**	6.292	6.269	0.02	6.303	−0.01
**28**	6.398	6.288	0.11	6.404	−0.01
**29**	6.569	6.604	−0.04	6.605	−0.04
**30**	6.553	6.601	−0.05	6.534	0.02
**31**	6.469	6.539	−0.07	6.403	0.07
**32**	7.000	6.984	0.02	6.964	0.04
**33**	7.000	6.921	0.08	6.904	0.10
**34** ^t^	6.569	6.244	0.32	6.539	0.03
**35**	6.796	6.835	−0.04	6.710	0.09
**36**	6.699	6.696	0.00	6.572	0.13
**37** ^t^	6.638	6.503	0.14	6.448	0.19
**38**	6.638	6.570	0.07	6.601	0.04
**39**	6.770	6.781	−0.01	6.902	−0.13
**40** ^t,b^	6.495	6.678	−0.18	6.910	−0.42
**41** ^t,b^	6.187	6.390	−0.20	6.718	−0.53

^t^ Test set compounds; ^a^ CoMFA outliers; ^b^ CoMSIA outliers.

**Table 4:** *q*^2^ and *r*^2^*_ncv_* values after several Y-randomization tests.

	CoMFA		CoMSIA	
Iteration	*q* ^2^	*r* ^2^ *_ncv_*	*q* ^2^	*r* ^2^ *_ncv_*
random_1	−0.927	0.263	−0.392	0.210
random_2	−0.111	0.321	−0.075	0.204
random_3	−0.348	0.543	−0.116	0.181
random_4	−0.315	0.427	−0.036	0.259
random_5	−0.192	0.343	−0.075	0.216
random_6	−0.192	0.332	−0.171	0.154
random_7	−0.303	0.361	−0.769	0.399
random_8	−0.051	0.345	−0.004	0.283
random_9	−0.262	0.374	−0.166	0.310
random_10	−0.001	0.339	−0.092	0.175

**Table 5:** The proposed structures of new molecules and their predicted pEC_50_ values using the best model.

Entry	Structure	Predicted pEC_50_
1x	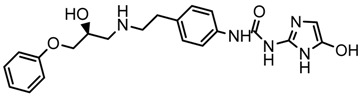	7.186
2x	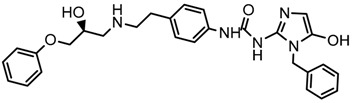	8.442
3x	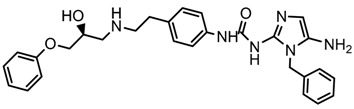	8.561
4x	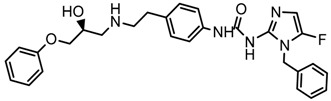	8.021
5x	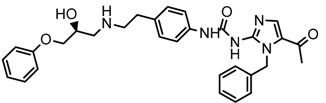	7.960
6x	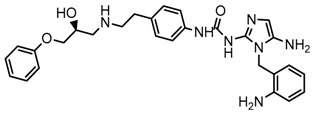	8.100
7x	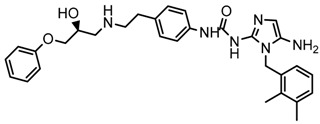	8.520
8x	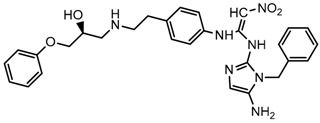	7.590
9x	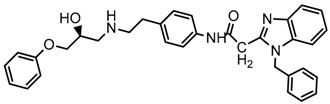	7.260
10x	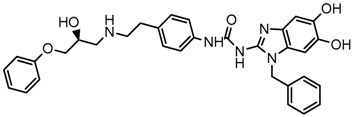	7.680
11x	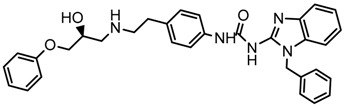	7.580
12x	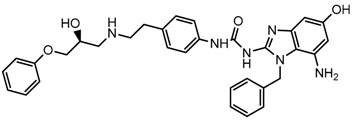	8.000

**Table 6:** Chemical structures and pEC_50_ values of the studied β3-adrenergic ligands ^a^.

Entry	Structure		EC_50_ (μM)	pEC_50_
	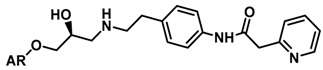			
1	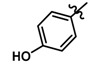		0.29	6.538
2	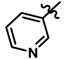		0.18	6.745
3	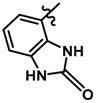		0.28	6.553
4	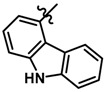		0.25	6.602
	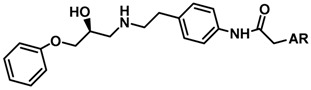			
5	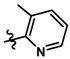		0.33	6.481
6	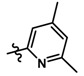		0.28	6.553
7	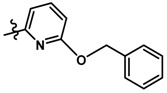		2.50	5.602
8	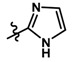		9.60	5.018
9	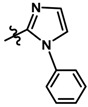		0.65	6.187
10	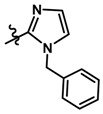		0.18	6.745
11	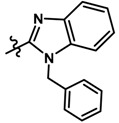		0.14	6.854
12	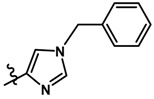		0.13	6.886
13	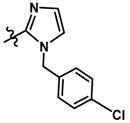		0.27	6.569
14	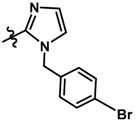		0.19	6.721
15	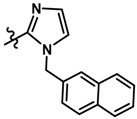		0.26	6.585
	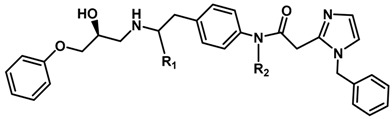			
	R_1_	R_2_		
16	(*S*)-Me	H	0.062	7.208
17	H	Me	0.28	6.553
18	H	Propyl	0.50	6.301
	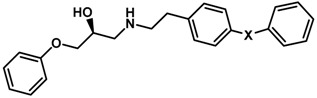			
19	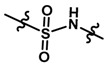		2.0	5.699
20	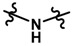		12.0	4.921
21	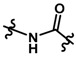		130	3.886
22	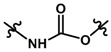		9.30	5.032
23	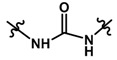		1.90	5.721
24	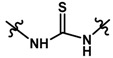		0.44	6.357
	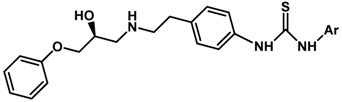			5.854
25	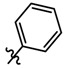		1.40	6.328
26	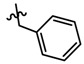		0.47	6.292
27	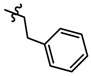		0.51	6.398
28	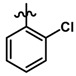		0.40	5.854
29	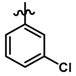		0.27	6.569
30	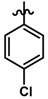		0.28	6.553
31	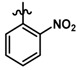		0.34	6.469
32	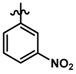		0.10	7.000
33	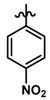		0.10	7.000
34	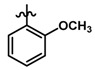		0.27	6.569
35	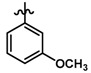		0.16	6.796
36	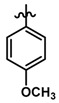		0.20	6.699
37	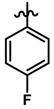		0.23	6.638
38	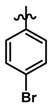		0.23	6.638
39	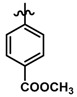		0.17	6.770
40	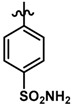		0.32	6.495
41	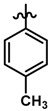		0.65	6.187

^a^ EC_50_ = Half maximal effective concentration; pEC_50_ = −logEC_50_; M = molL^−1^.

## Supplementary Materials

The following are available online, Figure S1: Distill-based alignment of optimized molecules by Powell method, Table S1: q2 and N values for all field combinations of CoMFA and CoMSIA, Table S2: Randomizations of biological activity for the execution of the Y-random test, Table S3: Statistical parameters for CoMFA and CoMSIA based in the Powell minimization method
