# Supplementary material for: Structure-Activity Relationships Based on 3D-QSAR CoMFA/CoMSIA and Design of Aryloxypropanol-Amine Agonists with Selectivity for the Human β3-Adrenergic Receptor and Anti-Obesity and Anti-Diabetic Profiles
Source: Molecules. 2018 May 16;23(5):1191. doi: 10.3390/molecules23051191 (PMC6099677; doi:10.3390/molecules23051191)
Supplement: Supplementary file 1 [file molecules-23-01191-s001.pdf]

Supplementary Material

Structure-Activity Relationships Based on 3D-QSAR CoMFA/CoMSIA and Design of Aryloxypropanol-amine Agonists with Selectivity for the Human β3-Adrenergic Receptor and Anti-Obesity and Anti-Diabetic Profiles

Marcos Lorca [1], Cesar Morales-Verdejo [2], David Vásquez-Velásquez [3], Juan Andrades-Lagos [3], Javier Campanini-Salinas [4], Jorge Soto-Delgado [5], Gonzalo Recabarren-Gajardo [6] and Jaime Mella [7,8]*

1. Escuela de Quimica y Farmacia, Facultad de Medicina, Universidad Andres Bello, Quillota 980, Viña del Mar 2531015, Chile; m.lorcacarvajal@uandresbello.edu
2. Centro de Nanotecnología Aplicada, Facultad de Ciencias, Universidad Mayor, Camino la Pirámide 5750, Huechuraba, Santiago 8580000, Chile; camoralv@uc.cl
3. Facultad de Ciencias Químicas y Farmacéuticas, Universidad de Chile, Sergio Livingstone 1007, Independencia, Santiago 8380492, Chile; dvasquez@ciq.uchile.cl (D.V.-V.); jandrades@ug.uchile.cl (J.A.-L.)
4. Facultad de Ciencia, Universidad San Sebastián, Lago Panguipulli 1390, Puerto Montt 5501842, Chile; javier.campanini@uss.cl
5. Departamento de Ciencias Quimicas, Facultad de Ciencias Exactas, Universidad Andres Bello, Quillota 980, Viña del Mar 2531015, Chile; jorge.soto@unab.cl
6. Departamento de Farmacia, Facultad de Química, Pontificia Universidad Católica de Chile, Casilla 306, Avda. Vicuña Mackenna 4860, Macul, Santiago 7820436, Chile; grecabarren@uc.cl
7. Centro de Investigación Farmacopea Chilena (CIFAR), Universidad de Valparaíso, Av. Gran Bretaña 1111, Valparaíso 2360102, Chile
8. Instituto de Química y Bioquímica, Facultad de Ciencias, Universidad de Valparaíso, Av. Gran Bretaña 1111, Valparaíso 2360102, Chile

* Correspondence: jaime.mella@uv.cl; Tel.: +56-032-250-8067

Table S1. q^2 and N values for all field combinations of CoMFA and CoMSIA.[a]

**Model**	**q^2**	**N**
CoMFA-S	0.442	3
CoMFA-E	0.331	12
CoMFA-SE	0.537	6
CoMSIA-S	0.293	2
CoMSIA-E	0.135	2
CoMSIA-H	-0.071	5
CoMSIA-D	-0.024	2
CoMSIA-A	-0.014	2
CoMSIA-SE	0.566	7
CoMSIA-SEH	0.669	20
CoMSIA-SEHD	0.654	20
CoMSIA-SEHA	0.674	6
CoMSIA-SED	0.66	16
CoMSIA-SEA	0.651	5
CoMSIA-SEDA	0.601	7
CoMSIA-SH	0.464	7
CoMSIA-SD	0.551	6
CoMSIA-SA	0.409	4
CoMSIA-SHD	0.561	9

Table S1. *Cont.*

CoMSIA-SHA	0.403	8
CoMSIA-SDA	0.622	15
CoMSIA-SHDA	0.613	20
CoMSIA-EH	0.536	20
CoMSIA-ED	0.349	2
CoMSIA-EA	0.48	3
CoMSIA-EHD	0.422	5
CoMSIA-EHA	0.598	6
CoMSIA-EDA	0.328	2
CoMSIA-EHDA	0.508	7
CoMSIA-HD	0.02	6
CoMSIA-HA	0.004	20
CoMSIA-HDA	0.107	20
CoMSIA-DA	-0.062	1
CoMSIA-ALL	0.669	6

[a] q^2 = the square of the LOO cross-validation (CV) coefficient; N = the optimum number of components.

Table S2. Randomizations of biological activity for the execution of the Y-random test.

Random-1	Random-2	Random-3	Random-4	Random-5	Random-6	Random-7	Random-8	Random-9	Random-10
6.4685	7.2076	6.4685	6.2924	6.8861	6.4685	6.3979	6.4815	5.6021	6.585
4.9208	6.2924	6.4815	6.7447	7	6.8861	5.6021	6.3565	7.2076	6.3979
6.3565	6.7959	5.0315	7.2076	5.8539	6.7959	6.5528	6.5528	6.7447	6.8861
6.6383	6.3565	6.2924	6.6383	6.6383	5.8539	5.8539	5.0315	6.5528	5.6021
7.2076	6.5528	6.5528	4.9208	6.5686	6.5686	4.9208	6.6383	6.5686	6.5528
6.5528	6.3979	6.5686	6.5528	6.585	6.5528	6.5686	6.5686	6.5528	6.7447
5.6021	6.7696	6.6383	5.8539	6.3279	6.1871	6.5528	6.8861	6.6383	6.3279
3.8861	7	6.585	6.3565	6.1871	7.2076	6.4685	6.699	5.0315	6.1871
6.3979	6.5686	6.5528	7	7.2076	6.7696	6.7447	6.3279	6.1871	6.7959
7	6.3279	3.8861	6.5528	6.4685	6.7447	7	6.6021	6.3565	6.699
6.7447	6.5686	6.5686	6.4815	6.6021	4.9208	5.699	6.2924	6.5528	6.5686
7	6.5528	5.699	6.4685	6.2924	7	7	6.7696	6.585	5.8539
5.0315	6.5528	5.6021	6.7696	7	6.699	6.7959	6.1871	6.4815	6.6383
6.4815	6.7447	6.7696	6.699	6.5528	6.6021	6.5686	7.2076	6.3279	6.2924
6.5528	5.699	4.9208	6.3279	6.7696	6.5686	6.6021	4.9208	7	6.5686
6.7959	4.9208	6.1871	6.585	6.7447	6.4815	7.2076	7	5.8539	6.7696
6.5686	3.8861	7	5.0315	4.9208	6.3565	6.2924	6.585	6.4685	6.5528
6.699	6.8861	7.2076	6.5686	6.3565	6.6383	6.585	6.4685	7	6.5528
6.3279	5.8539	6.3279	7	6.699	5.0315	6.3565	6.5528	6.699	7
5.8539	6.4815	6.7959	6.3979	6.3979	6.3279	6.4815	6.7447	6.7696	6.6021
6.2924	6.699	6.3565	6.7959	6.5528	6.5528	6.1871	6.7959	6.3979	5.699
6.7696	6.6021	6.8861	5.699	5.699	6.5528	6.7696	7	6.5686	7.2076
5.699	7	7	6.5686	6.5528	6.2924	6.5528	6.5686	6.7959	7
6.5686	6.6383	6.699	5.6021	6.4815	6.3979	6.3279	5.8539	5.699	6.4685
6.1871	5.6021	6.6021	6.6021	6.5686	6.585	6.699	5.699	3.8861	6.3565
6.5528	6.1871	5.8539	6.8861	5.0315	3.8861	3.8861	6.5528	6.8861	6.4815
6.8861	6.585	6.5528	3.8861	6.7959	5.699	6.8861	6.3979	4.9208	3.8861
6.6021	6.4685	6.7447	6.5528	5.6021	7	6.6383	5.6021	6.6021	5.0315
6.585	5.0315	6.3979	6.1871	3.8861	5.6021	5.0315	3.8861	6.2924	4.9208

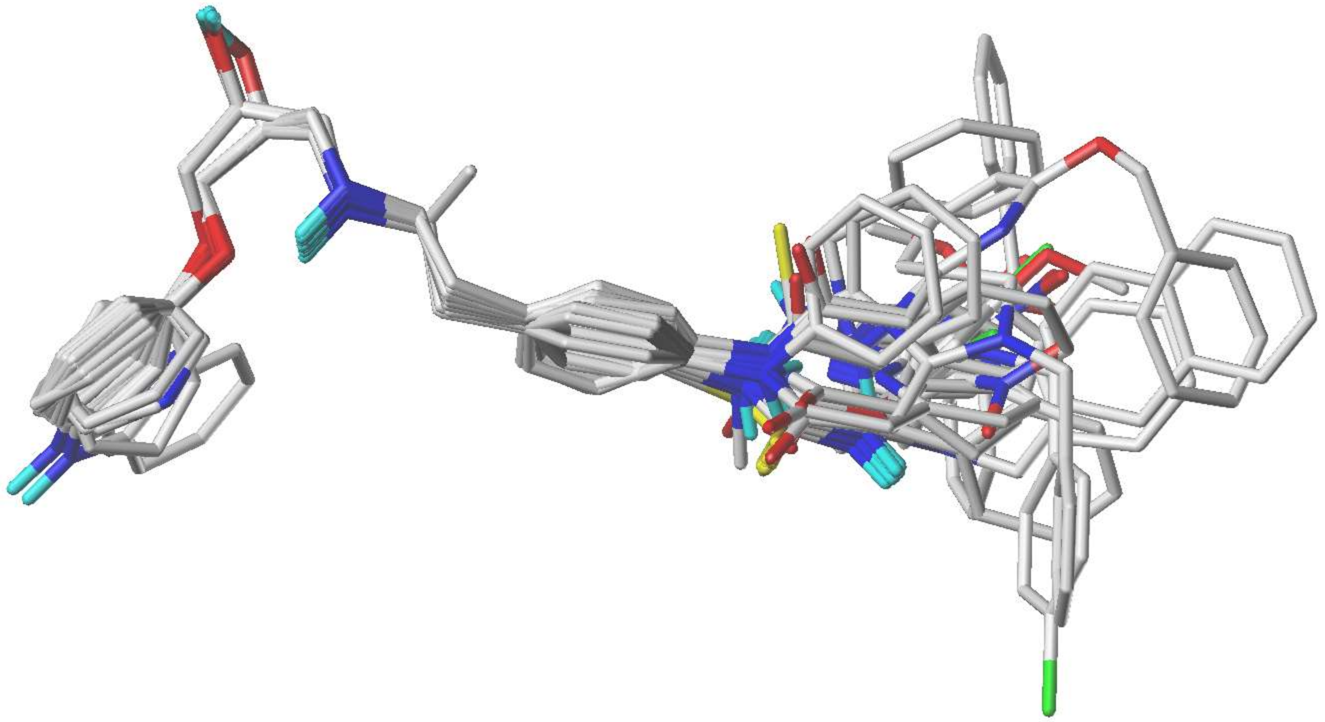

Figure S1. Distill-based alignment of optimized molecules by Powell method.

Table S3. Statistical parameters for CoMFA and CoMSIA based in the Powell minimization method.[a]

Parameter	CoMFA	CoMSIA
q^2	0.176	0.12
N	1	2
SEP	0.655	0.69
SEE	0.469	0.413
r^2	0.577	0.685
F	36.8	28.3
S	0.435	0.130
E	0.565	0.258
H	-	0.120
D	-	0.257
A	-	0.235

[a] q^2 = the square of the LOO cross-validation (CV) coefficient; N = the optimum number of components; SEP = standard error of prediction; SEE is the standard error of estimation of non CV analysis; r^2 is the square of the non CV coefficient; F is the F-test value; S, E, H, D and A are the steric, electrostatic, hydrophobic, hydrogen-bond donor, and hydrogen-bond acceptor contributions respectively.
